# Aerosol Jet Printing of 3D Pillar Arrays from Photopolymer Ink

**DOI:** 10.3390/polym14163411

**Published:** 2022-08-20

**Authors:** Vitor Vlnieska, Evgeniia Gilshtein, Danays Kunka, Jakob Heier, Yaroslav E. Romanyuk

**Affiliations:** 1 Empa—Swiss Federal Laboratories for Materials Science and Technology, Überlandstrasse 129, 8600 Dübendorf, Switzerland; 2 Institute of Microstructure Technology (IMT), Karlsruhe Institute of Technology (KIT), Hermann-von-Helmholtz-Platz 1, 76344 Eggenstein-Leopoldshafen, Germany

**Keywords:** photopolymer, photoresin, aryl epoxy oligomers, aerosol jet printing, reactive ion etching, 3D structures, microfabrication

## Abstract

An aerosol jet printing (AJP) printing head built on top of precise motion systems can provide positioning deviation down to 3 μm, printing areas as large as 20 cm × 20 cm × 30 cm, and five-axis freedom of movement. Typical uses of AJP are 2D printing on complex or flexible substrates, primarily for applications in printed electronics. Nearly all commercially available AJP inks for 2D printing are designed and optimized to reach desired electronic properties. In this work, we explore AJP for the 3D printing of free-standing pillar arrays. We utilize aryl epoxy photopolymer as ink coupled with a cross-linking “on the fly” technique. Pillar structures 550 μm in height and with a diameter of 50 μm were 3D printed. Pillar structures were characterized via scanning electron microscopy, where the morphology, number of printed layers and side effects of the AJP technique were investigated. Satellite droplets and over-spray seem to be unavoidable for structures smaller than 70 μm. Nevertheless, reactive ion etching (RIE) as a post-processing step can mitigate AJP side effects. AJP-RIE together with photopolymer-based ink can be promising for the 3D printing of microstructures, offering fast and maskless manufacturing without wet chemistry development and heat treatment post-processing.

## 1. Introduction

### Aerosol Jet Printing—AJP

In a period of almost two decades, AJP has been consistently utilized in the field of printed electronics and bio-applications [1]. In the 2000s, due to significant advances in hardware and software for motion systems technology, commercial AJP models such as the Aerosol Jet (Optomec Inc., Albuquerque, NM, USA) and the Nanojet systems (Integrated Deposition Solutions Inc.—IDS, Albuquerque, NM, USA) were released [2]. These AJP equipment can reach printing resolution down to a few micrometers regarding line width, offering advantages of fast prototyping and high-volume manufacturing [3,4], being a maskless technique and non-contact printing process [5], and along with it, one can directly convert CAD (Computer-Aided Design) diagrams to CAM (Computer-Aided Manufacturing) tool paths (motion system actuation commands).

**Figure 1:**
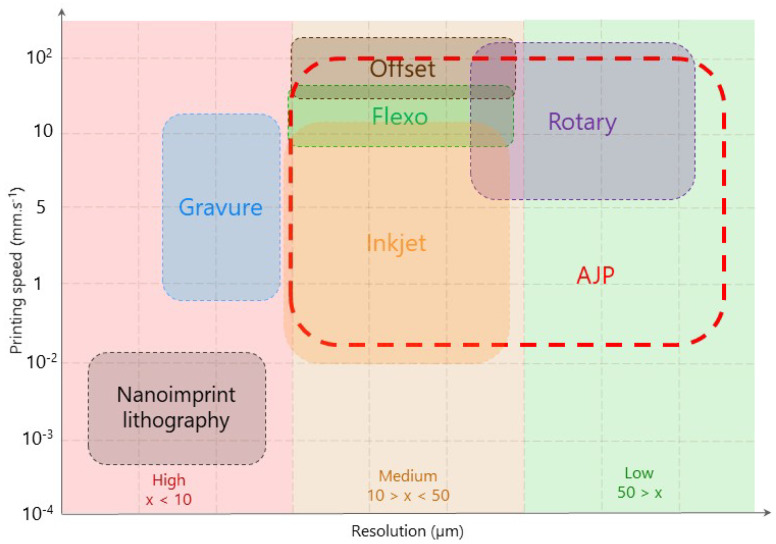
Printing speed and resolution of some popular printing techniques. (Adapted from Wu (2017) [7] with permission).

AJP is considered as a non-contact printing technique; nevertheless, some authors also classify AJP as the so-called Direct Write (DW) printing [6]. It became an essential tool when three-dimensional substrates are used, and printing resolution in the range of hundreds down to a few micrometers is needed. Figure 1 represents the speed and spatial printing resolution of popular printing techniques.

**Figure 2:**
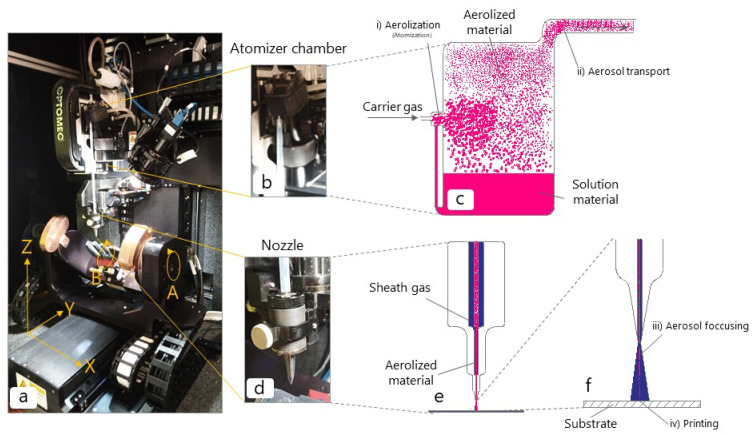
Aerosol jet printing. (**a**) AJ 5X equipment (Optomec—Albuquerque, US) with a 5-axis motion system. (**b**) Ultrasonic atomizer chamber. (**c**) Aerosolization process. (**d**) Printing nozzle. (**e**) Representation of an aerolized material and sheath gas inside the nozzle path. (**f**) Focused aerosolized material.

Figure 1 presents printing techniques such as gravure, screen, inkjet, flexographic and aerosol, showing the flexibility of applying different substrates, ink formulation and ink composites at different process parameters [8]. They do not require the usage of harmful chemicals and do not produce material waste [9]. Specifically, regarding AJP, one can observe the versatility of this technique, where resolution can be tuned from 10 to hundreds of micrometers line width, as well as speed printing varying from 1.0 × 10^−2^ up to 1.5 × 10^2^ mm · s^−1^. Comprehensive reviews of AJP were presented by Wilkinson (2019) [1], Secor (2018) [8], Binder et al. (2014) [10], Hines et al. (2021) [6], and Jabari et al. (2016) [11]. The AJP process can be briefly summarized in the following four main steps [12] (see also Figure 2):

(i) Ink aerosolization;
(ii) Aerosol transport by a carrier gas;
(iii) Aerosol focusing;
(iv) Material transfer to the substrate.

As shown in Figure 2 (i), utilizing the ultrasonic atomization method, small volumes of solution (usually 2 to 3 mL) can be aerosolized, resulting in dispersed droplets of diameter size ranging from 2 to 5 μm. Atomization parameters and ink formulation have to be considered in order to reduce satellite droplets and over-spray effects during the printing section [1]. In the Figure 2 second step (ii), a carrier gas (usually nitrogen) is applied to transport aerosol to the tip nozzle, where controlled droplet diameter sizes can be achieved [10]. In step (iii), focusing of the aerosol is accomplished by the application of a parallel and cylindrical gas flow (sheath flow) of aerosol boundaries. The ratio between aerosol and sheath flow rates is known as the focus ratio [13], which is one of the major optimization parameters. In step (iv), aerosol is transferred to the substrate with typical distances between the nozzle tip and substrate ranging from 2 to 5 mm. Methodologies to optimize AJP process parameters were discussed by Mahajan et al. (2013) [13], Phuah et al. (2020) [14], Catic et al. (2020) [15], Chen et al. (2018) [16], Goth et al. (2011) [17], Seifert et al. (2015) [18], Smith et al. (2017) [19], and Williams et al. (2020) [20]. The main purpose of these works is to show how to achieve desired features such as line width and thickness of the jetting ink, and simultaneously to it, reducing as much as possible satellite droplets and over-spray surrounding the printing path. In order to reach a stable and optimized line width, it is necessary to consider a significant number of process variables together with the material’s chemistry. Phuah et al. (2020) [14], Manhajan et al. (2013) [13], and Goth et al. (2011) [17] reported a diverse set of variables that affect the printing quality, where the most critical ones are listed in Table 1.

In parallel to process and ink parameters optimization, the tool path (or motion path) has to be concisely considered to achieve ideal printing quality. The tool path is a CAD pattern converted to a motion programming language (in our case, ACSPL+), resulting in the printing path. Within the printing path, it is worth observing geometries of small angle vertices, which usually leads to bulky regions of ink deposition, where one has to either accept this side effect or compromise with the printing design. The main advantages of AJP are the considerable distance of the tip nozzle to the substrate and freedom of design pattern, allowing to print on complex three-dimensional substrates [21]. These major advantages allow an easy hybridization of fabrication processes and facilitate the integration and connection of the manufactured parts [1]. Due to the large range of parameters specifications, applications ranging from novel electronics design [12], radio frequency (RF) engineering research [22], electrical chip interconnections [21], microfluidic devices [23], patterning of biomaterials such as proteins, DNA fragments [2] and cell suspension [3], carbon nanotubes (CNTs) for thin-film transistors (TFTs) [24], CNTs for top-gate field-effect transistors [25], organic light-emitting diodes (OLEDs) [26], thin film growth for perovskites solar cells [27], front side panel layers for silicon solar cells [28], in the field of carbon technology by means of interconnects using graphene ink [11,29], microfluidic sensors for biosensing application [23], and high-sensitivity ammonia sensor [30] are some of the extensive applications of AJP recently reported in the literature. To date, a 10 μm line width is the smallest resolution achieved with AJP, being printed utilizing conductive silver ink [1].

When working within AJP’s resolution limit (from 70 down to 10 μm), satellite droplets and over-spray in printing path boundaries are strongly pronounced. An in-depth study considering the focus ratio, substrate temperature and its nature is presented by Smith et al. (2017) [19]; furthermore, Chen et al. (2019) [16] reported a model simulation based on fluid dynamics to optimize AJP process parameters. Nonetheless, one can observe that even for optimal AJP printing conditions, satellite droplets were present in printed samples.

It is worth noting that the majority of AJP applications are 2D printing on complex or flexible substrates, primarily for printed electronics applications. Regarding AJP to manufacture 3D structures, to date, the literature presents few studies in which three-dimensional structures have been previously realized by a hybrid process of AJP and micro stereolithography, resulting in structures of a high aspect ratio (HAR) of approximately 1 [31] as well as hybrid AJP and direct laser writing (DLW) for 2D patterning [32]. HAR structures produced through point-wise spatial printing (a similar approach of AJP) were reported by Saleh (2017) [33]. Nevertheless, if AJP could be realized as an additive manufacturing (AM) process, applications such as optical components for imaging analysis based in multi-contrast techniques and high adhesion geko-like structures [34] could be manufactured without the need of a mask, and the printing of larger areas can be achieved. In addition, AJP as AM can overcome the challenge of 3D printing microstructures on top of three-dimensional substrates.

In this work, we explore AJP as a 3D additive manufacturing technique, where pillar structures of 550 μm height and a diameter of 50 μm were manufactured. Photopolymer based in aryl epoxy resin and a cationic photoinitiator was formulated to achieve photo-sensitivity at 365 nm and together with the strategy of cross-linking the material during printing (curing on the fly), it was possible to manufacture 3D structures. In addition to exploring the AJP 3D manufacturing technique, we present a manufacturing process combining AJP and reactive ion etching (RIE) as a post-processing step toward the mitigation of AJP satellite droplets and over-spray side effects.

Applications such as optical components for imaging analyses based on multi-contrast techniques and high-adhesion geko-like structures could benefit from this outcome.

## 2. Materials and Methods

### 2.1. Materials

Aryl epoxy oligomers (poly(2,2-bis(4-oxy-(2-(methyloxirane)phenyl)propan) of low averaged molar mass (607 Da), 1.015 polidispersity, and epoxidation degree of 36% mol · mol${}_{polymer}^{-1}$ were previously obtained and characterized following the procedure described by Vlnieska et al. (2019) [35]. Triarylsulfoniumhexafluorantinonat salts (TAS) (50 wt % in propylene carbonate) and polar solvents such as ketones and isopropanol (IPA) (anhydrous, 99.5%), were utilized for the photopolymer formulation. Chemicals and consumables were purchased from Sigma-Aldrich (Darmstadt, Germany) and used as received.

### 2.2. Photopolymer Formulation

Aryl epoxy oligomers (poly(2,2-bis(4-oxy-(2-(methyloxirane)phenyl)propan) were diluted in cyclopentanone in a ratio of 92 % mol · mol${}_{polymer}^{-1}$, and TAS was added in a ratio of 3 % mol · mol${}_{polymer}^{-1}$. Viscosity of 11 cP was obtained.

### 2.3. Aerosol Jet Printing

Structures were printed using an Aerosol Jet Flex AJ5X equipment (Optomec— Albuquerque, NM, USA). An ultrasonic atomization module was used to generate the aerosol. Utilized parameter values were (values in standard cubic centimeters per minute—SCCM): atomizer pressure: 25; sheath flow pressure: 110; virtual impact pressure: 2; substrate stage heating: 75 °C; ultrasonic current: 500 mA, 365 nm; LED array: 2.8 mW · cm^−2^ with light intensity of 90%.

### 2.4. Reactive Ion Etching (RIE)

Three-dimensional (3D) structures were post-processed using a STP2020 equipment from R3T GmbH (Taufkirchen, Germany). Oxygen (O_2_), Tetrafluormethane (CF_4_) and Nitrogen (N_2_) were kept at 450 mTorr, the system was operated at 1200 W, and processing temperature was kept at 22 °C.

### 2.5. Scanning Electron Microscopy

Measurements were carried out using a microscope S4700 (Hitachi—Tokio, Japan). The beam energy was 10 keV, the current was 3 mA, and a low-resolution imaging mode was used to acquire the images.

## 3. Results and Discussion

### 3.1. Photopolymer Design

Ink formulation is to date one of the key factors to achieve high-quality printing sections by aerosol jetting technology, in which three main composition properties have to be considered: (i) viscosity, (ii) maximum solid loading, (iii) maximum particle size. Recommended viscosity values are 6 to 12 cP for the ultrasonic atomization. Solid loading of 55 wt % is suggested as the maximum content for AJP ink formulations, and maximum particle sizes are 50 nm for ultrasonic and 500 nm for pneumatic atomization methods [1].

**Figure 3:**
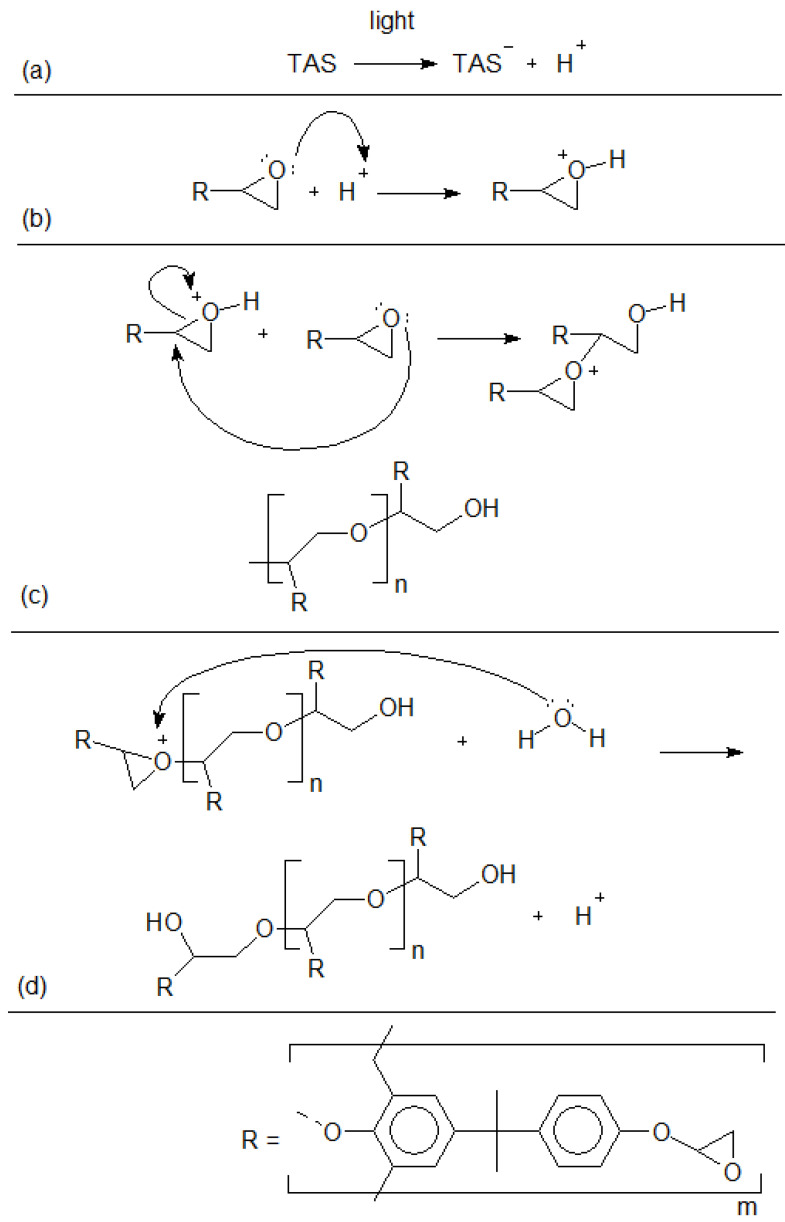
Aryl epoxy resin probable cross-linking reaction mechanism. (**a**) photoinitiator reaction with light. (**b**) epoxy group activation. (**c**) crosslinking reaction through epoxy groups. (**d**) epoxy group deactivation.

We have chosen aryl epoxy resins for the ink design due to a set of advantages. Epoxy groups from this polymer class present efficient reactivity with photo cationic initiators [35]. After light exposure, the TAS photoinitiator releases protons into the medium, initiating the reaction between epoxy groups. Figure 3 depicts the probable cross-linking reaction mechanism.

As shown in Figure 3a, through light exposure, the TAS photoinitiator releases protons into the medium. Afterward, oxygen reacts with protons, activating epoxy groups, as seen by Figure 3b. The cross-linking initiation and propagation processes are depicted in Figure 3c, where a second epoxy group reacts at tertiary carbon from the previous epoxy, opening and stabilizing it. This reaction propagates as far as epoxy groups surround activated oxygen atoms. In Figure 3d, termination of the cross-linking reaction is shown with a Lewis base, which regenerates protons to the medium. In this case, a water molecule is depicted. R is the photopolymer chain as depicted in the end of Figure 3.

**Figure 4:**
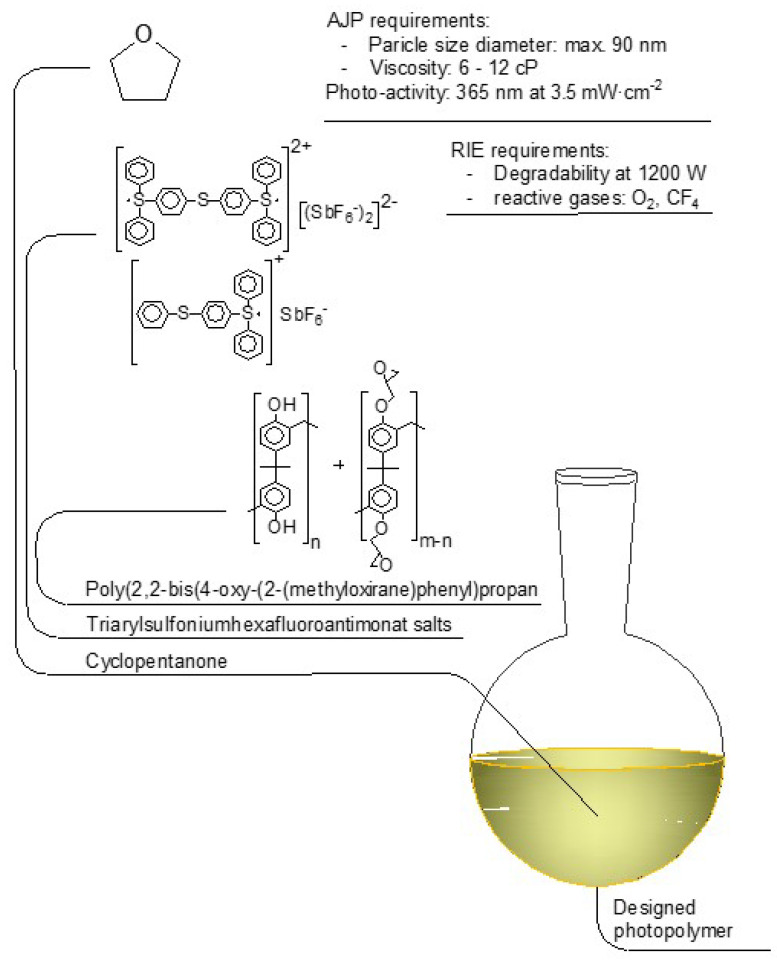
Photopolymer formulation. Aryl epoxy oligomer, TAS, and cyclopentantone chemical structures. AJP and RIE requirements for ink formulation.

In this experimental setup, a 365 nm LED array was utilized as a UV light source. Formulation ratios between the solvent, photopolymer and photoinitiator were previously investigated by Vlnieska et al. (2020) [36] using a similar light source; the formulation was slightly adapted to the AJP process. Not least important, this class of material has strong adhesion with a wide range of substrates, slow degree of shrinkage, high chemical stability and strong hardness after cross-linking reaction [37]. Finlay, utilizing aryl epoxy resins as AJP ink results in two additional advantages, since the photopolymer is soluble in cyclopentanone, and there is no need of adding extra chemicals. This formulation does not require the tuning of composition properties regarding solid loading and particle size. Figure 4 depicts the chemical structure of AJP ink components together with formulation requirement parameters for AJP and RIE.

Photo-resin’s degradability is known to be efficient from previous investigations with RIE post-processing at 1200 W, applying oxygen and carbon tetrafluoride as reactive gases and nitrogen as a carrier/purging gas [35,36].

### 3.2. AJP Parameters Optimization

AJP allows the utilization of a wide variety of inks, and it is possible to tune several process parameters, providing significant robustness to be utilized in a broad set of applications. Nevertheless, some authors might consider AJP robustness as a drawback [8]. Process parameters optimization is sensitive to the ink formulation and vice versa, which usually leads to a time-consuming activity. Concomitant to it, stability and reproducibility in long time printing sections are core challenges to be overcome during the optimization process. It is worth noting that commonly used optimal parameters for a certain ink do not transfer to new formulations, resulting in an optimization process from the beginning. In general, there are two main steps in the AJP parameter optimization process, where first parameter values are tuned to achieve aerosolized solution in the atomization chamber. In a second step, one needs to tune process parameters to adjust the jetting ink at the nozzle tip region. Usually, the second step is performed accessing the line width of a monolayer printed line [1,5,6,8,10,13,16], as depicted by Figure 4a–c.

**Figure 5:**
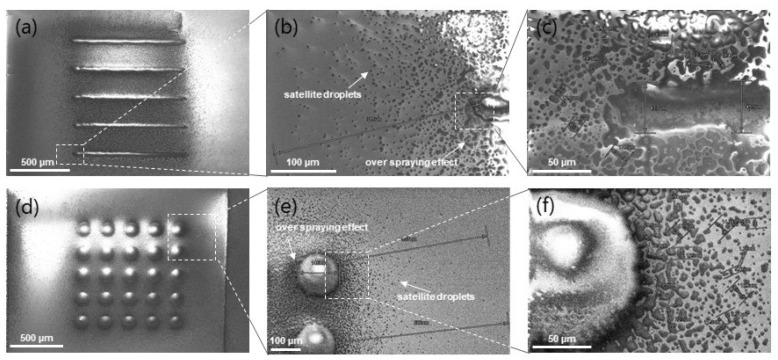
Top view of printed mono-layers. (**a**) Line and (**d**) base of pillar structures, (**b**,**e**) farthest droplets from the printing path, and (**c**,**f**) over-spray effect.

**Figure 6:**
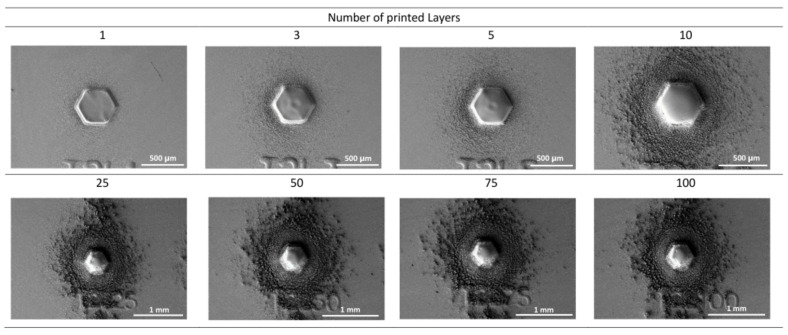
Over-spray effect depending on the number of printed layers.

Figure 5 presents over-spray and side droplets for line and array design; in (a), the line width achieved was 44.6 μm, in Figure 5b, the over-spray effect was at the farthest distance of 315 μm from the printing path, and in Figure 5c, one can note a considerable amount of side-droplets varying from 2 to 25 μm. When printing within AJP limit resolution (from 70 down to 10 μm line width), a pronounced over-spray and side droplet effects are expected at the substrate surface, even after parameters optimization [13,16,19]. In Figure 5d–f, one can observe similar side effects with pillar design. Consequently, for multi-layer printing, the accumulation of side droplets and over-spray at the substrate’s surface is anticipated. Figure 6 depicts micrographs from mono to hundred printed layers.

**Figure 7:**
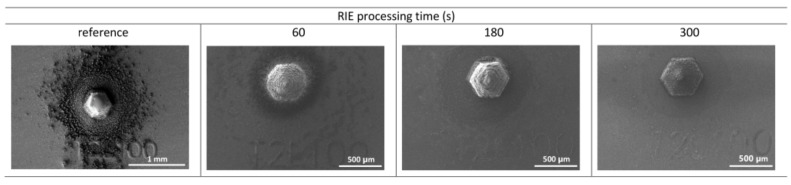
Removal of the over-spray by RIE with different processing times for a pillar with 100 printed layers.

As shown in Figure 6, for larger structures of approximately 500 μm in diameter, the over-spray effect is negligible up to printing three layers. Up to five printed layers, the AJP side effects begin to be pronounced regardless of the size of the structures. Usually, excessive ink material dries while printing and reaches the substrate surface in a solid state, making it difficult to accomplish a complete cross-linking reaction. An approach to mitigate AJP side effects is to apply reactive ion etching (RIE) as a post-processing step. As our photopolymer ink is known to be efficiently responsive to the RIE technique, etching conditions were investigated. Figure 7 depicts results for the optimal RIE parameters, increasing the etching time.

Figure 7 presents the highest printed pillar structure from the top view. One can observe the mitigation of AJP side effects starting at 60 s etching time. Up from 180 s etching time, most of the over-spray and satellite droplets were removed from the substrate surface. Figure S1 in the supplementary information presents the RIE process for the entire set of printed samples.

**Figure 8:**
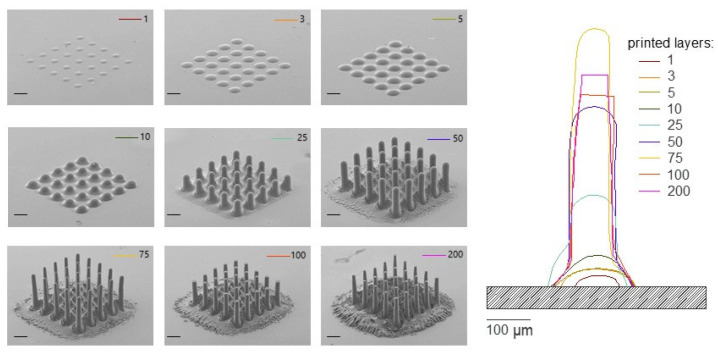
High aspect ratio pillar arrays with different numbers of printed layers ranging from 1 to 200, post-processed utilizing RIE. Height profiles of individual 3D pillars are presented on the right. Scale bar at images is 100 μm.

Figure 8 depicts the printing process of a pillar array along with an ascendant number of printed layers. The array design is 5 × 5 units, 100 μm pillar diameter and 125 μm distance between pillars.

In Figure 8 on the left side, one can observe pillar array micrographs from a 60 degrees view perspective. Printed layers ranged from 1 to 200. As expected, with the increase of printed layer number, AJP side effects are proportionally pronounced. Nonetheless, it is worth noting the effective substrate surface cleaning after applying RIE post-processing, mitigating most of these side effects in the external surface of the pillar arrays. Inside the pillar arrays and near the surroundings of the array, one can observe residual material after 25 printed layers. In Figure 8, on the right, one can observe the profile of the pillars. Interestingly, the etching ratio is more effective at the top of the pillars for the 100 and 200 printed layers structure, resulting in flat surfaces and reduced height when compared with 75 printed layers.

Heights of approximately 550 μm and 50 μm diameter on top of the pillars were achieved, resulting in an aspect ratio of 11.

## 4. Conclusions

This study explores AJP for the additive manufacturing of microstructures. In order to achieve 3D microstructures through AJP, photopolymer chemistry is a key factor to be considered. AJP ink was designed from an aryl epoxy resin and a cationic photoinitiator to be sensitive to the 365 nm UV light. The use of a photopolymer in combination with cross-linking “on the fly” enabled high aspect ratio pillar structures of 50 μm diameter and 550 μm in height, resulting in an HAR of 11.

When working at the AJP resolution limits, side effects such as over-spray and satellite droplets seem to be unavoidable. Reactive ion etching was investigated as post-processing to mitigate AJP side effects. For a large feature size of 500 μm in diameter, RIE can efficiently clean the substrate surface, whereas for the pillar microstructures, AJP over-spray could not be removed between pillars.

AJP-RIE together with photopolymer-based ink can be promising for the 3D printing of microstructures, offering fast and maskless manufacturing without wet development and heat treatment post-processing. Applications such as optical components for imaging analysis based on multi-contrast techniques and high adhesion gecko-like structures could benefit from this outcome. 

## Figures and Tables

**Table 1:** Principal AJP parameters.

Source	Variable	In This Work
Process	atomization gas flow sheath gas flow focus ratio aerosol flow temperature CAD design virtual impact flow	25 SCCM ^a^ 110 SCCM ^a^ 4.422 °C 2D pillar diagram none
Material	solvent:solid ratio (% mol · mol${}_{polymer}^{-1}$) ink temperature particle size ink viscosity	cyclopentanone:aryl epoxy resin 92% 35 °C no particles 11 cP
Machine	printing speed stage temperature working distance nozzle diameter atomizer type ultrasonic current	3 mm· s^−1^ 75 °C 4.7 mm 200 μm ultrasonic 500 mA

^a^ standard cubic centimeters per minute.

## Supplementary Materials

The following supporting information can be downloaded at: https://www.mdpi.com/article/10.3390/polym14163411/s1, Figure S1. Reactive ion etching vs. number of printed layers.
