# Supplementary material for: Aerosol Jet Printing of 3D Pillar Arrays from Photopolymer Ink
Source: Polymers (Basel). 2022 Aug 20;14(16):3411. doi: 10.3390/polym14163411 (PMC9412835; doi:10.3390/polym14163411)
Supplement: Supplementary file 1 [file polymers-14-03411-s001.zip › polymers-1883735-supplementary.pdf]

Filled hexagons design

		Reactive plasma cleaning thickness (µm)			
		0 (ref. sample)	1	3	5
Number of printed layers	1	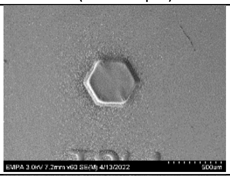	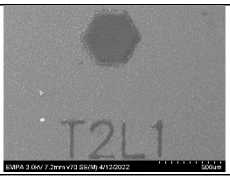	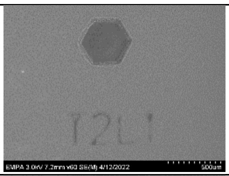	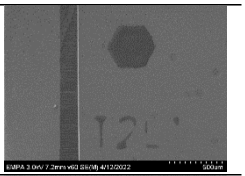
	3	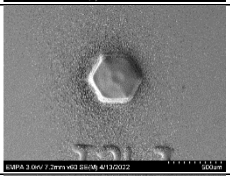	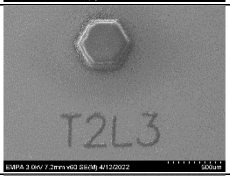	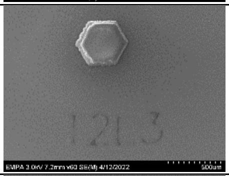	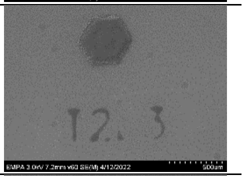
	5	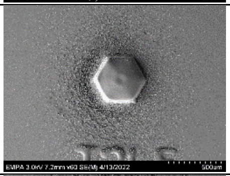	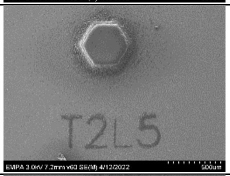	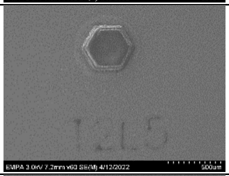	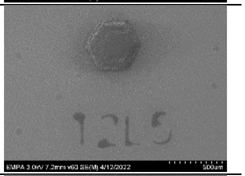
	10	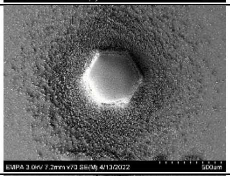	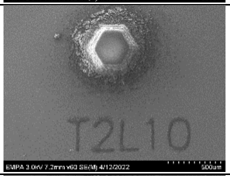	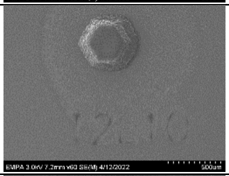	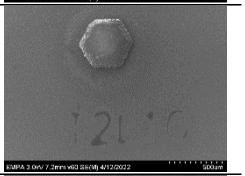
	25	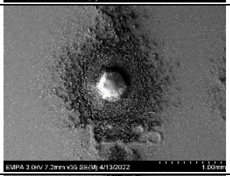	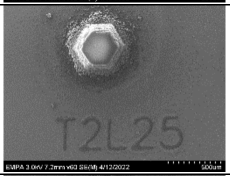	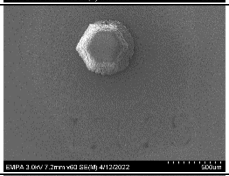	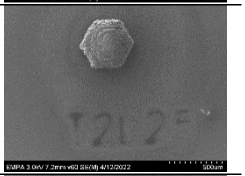
	50	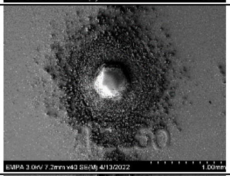	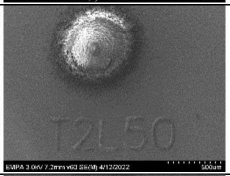	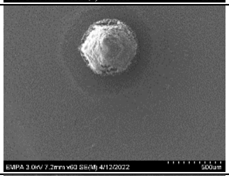	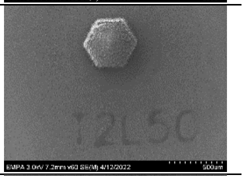
	75	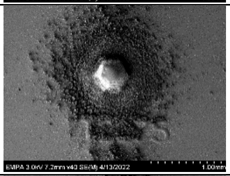	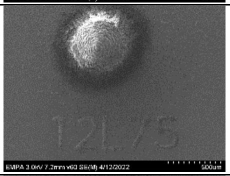	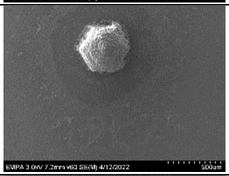	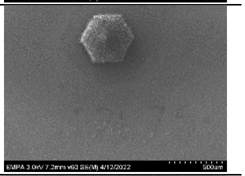
	100	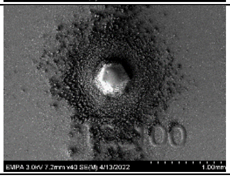	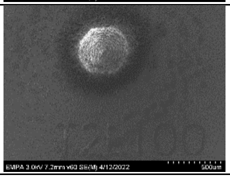	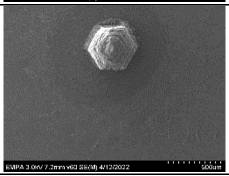	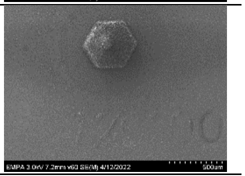

Hollowed hexagons design

		Reactive plasma cleaning thickness (µm)			
		0 (ref. sample)	1	3	5
Number of printed layers	1	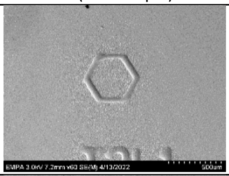	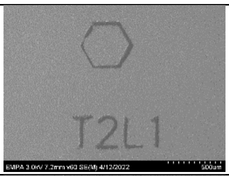	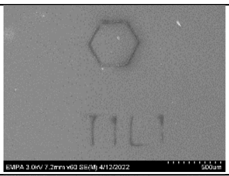	-
	3	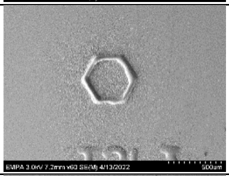	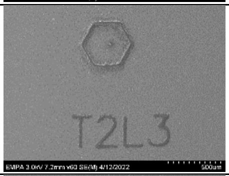	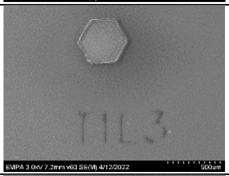	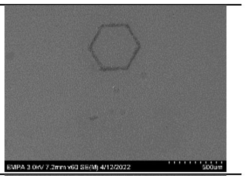
	5	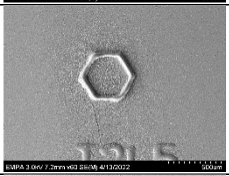	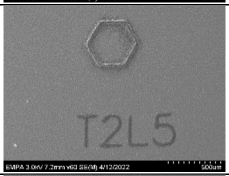	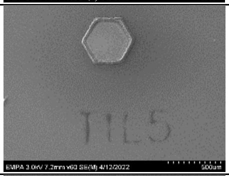	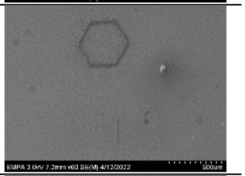
	10	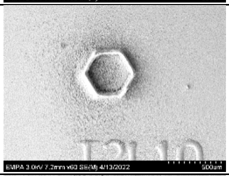	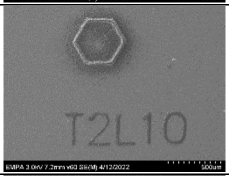	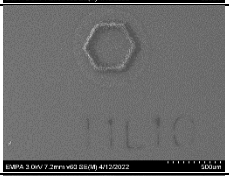	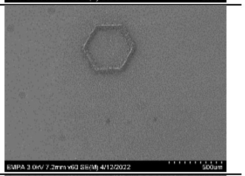
	25	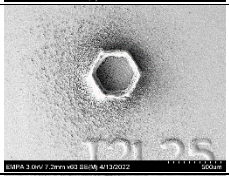	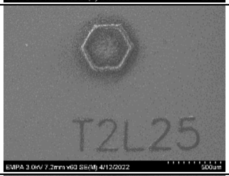	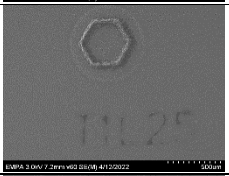	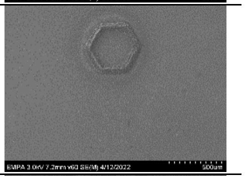
	50	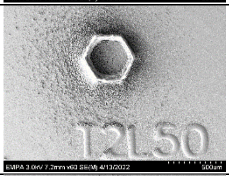	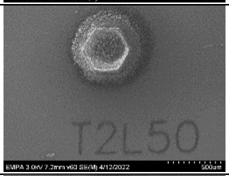	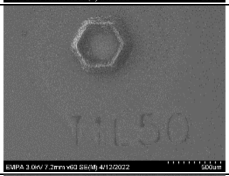	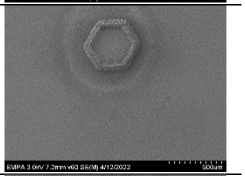
	75	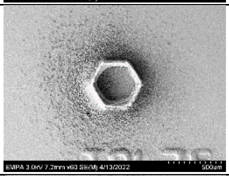	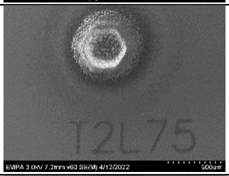	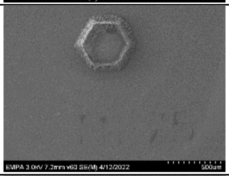	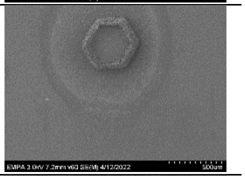
	100	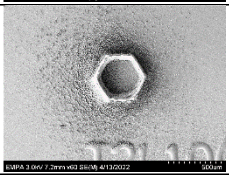	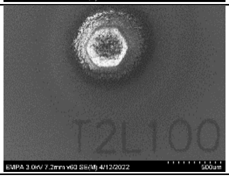	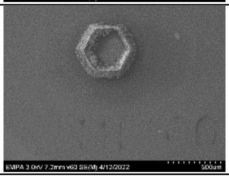	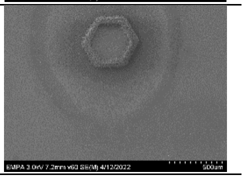

Filled hexagons design (height measurement*)			
Reactive plasma cleaning thickness (µm)			
		0 (ref. sample)	5
Number of printed layers	1	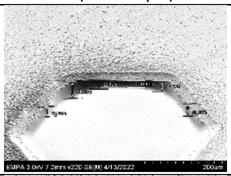	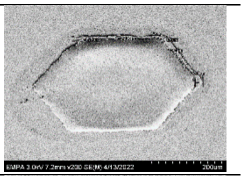
	3	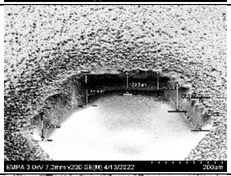	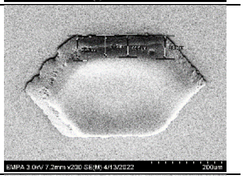
	5	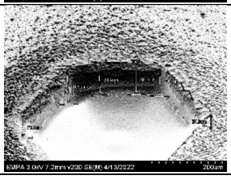	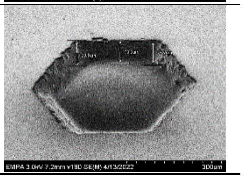
	10	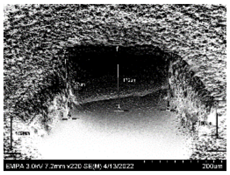	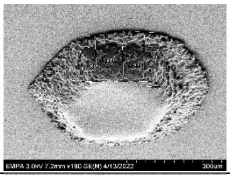
	25	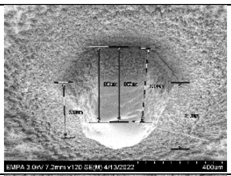	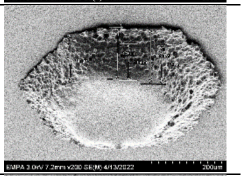
	50	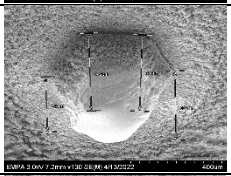	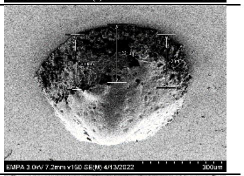
	75	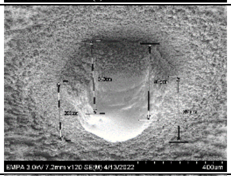	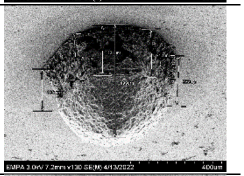
	100	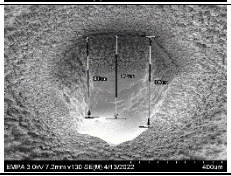	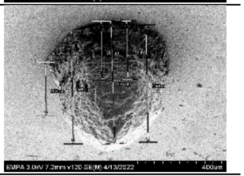

*SEM stage at 50 degrees inclination

Filled hexagons design (height measurement* averaged values)			
Reactive plasma cleaning thickness (µm)			
		0 (ref. sample)	5
Number of printed layers	1	22.5; 18.0; 26.2	12.9; 9.0; 11.3
	3	96.4; 69.5; 84.4	72.3; 64.5; 65.5
	5	96.4; 98.2; 89.4	80.5; 70.6; 77.2
	10	187; 172; 152	106; 118; 128
	25	360; 357; 350	141; 149; 158
	50	342; 336; 307	209; 221; 204
	75	348; 364; 314	201; 221; 212
	100	360; 372; 398	253; 281; 278

Hollowed hexagons design (height measurement*)			
Reactive plasma cleaning thickness (μm)			
		0 (ref. sample)	5
Number of printed layers	1	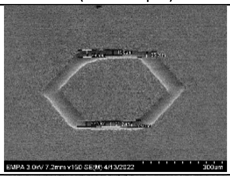	Structure totally removed
	3	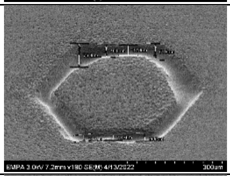	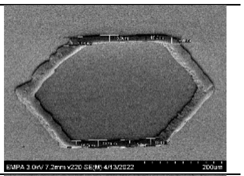
	5	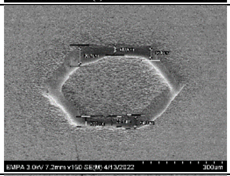	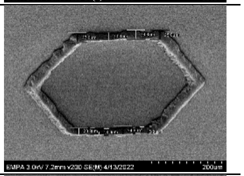
	10	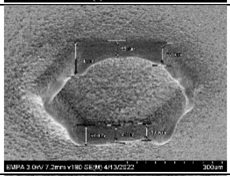	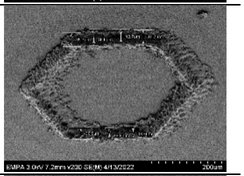
	25	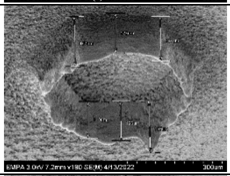	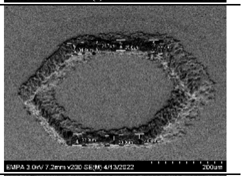
	50	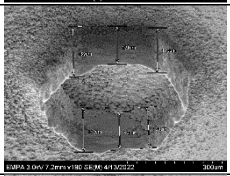	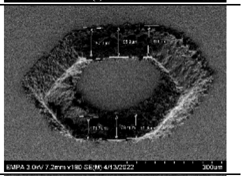
	75	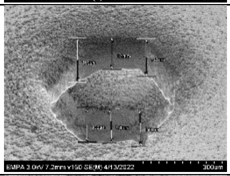	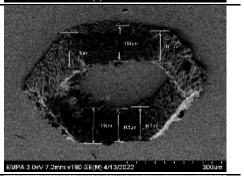
	100	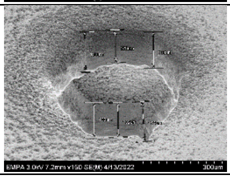	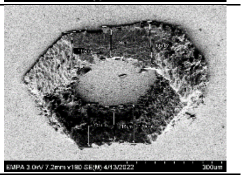

*SEM stage at 50 degrees inclination

**Hollowed hexagons design (height measurement* averaged values)**			
Reactive plasma cleaning thickness (µm)			
		0 (ref. sample)	5
Number of printed layers	1	26.5;19.8;25.2	-
	3	32.0;21.1; 26.5	20.8; 18.0; 15.3
	5	41.0; 38.4; 37.1	27.8; 23.8; 26.8
	10	55.1; 72.8; 68.3	39.7; 46.6; 40.7
	25	157; 127; 141	31.9; 31.7; 38.7
	50	159; 121; 143	97.0; 95.9; 100
	75	169; 122; 144	115; 114; 101
	100	153; 126; 138	142; 115; 126
